# Characterizing breed-shared and breed-specific genetic regulatory effects of gene expression across three pig breeds

**DOI:** 10.1186/s40104-026-01374-2

**Published:** 2026-04-09

**Authors:** Xiaojing Li, Xiaodian Cai, Jiajian Chen, Xiangchun Pan, Wentao Gong, Zhanming Zhong, Jinyan Teng, Zhe Zhang

**Affiliations:** State Key Laboratory of Swine and Poultry Breeding Industry, National Engineering Research Center for Breeding Swine Industry, Guangdong Provincial Key Lab of Agro-Animal Genomics and Molecular Breeding, College of Animal Science, South China Agricultural University, Guangzhou, 510642 Guangdong China

**Keywords:** Breed-stratified analysis, *Cis*-eQTL mapping, Complex traits, Gene expression, Regulatory architecture

## Abstract

**Background:**

Differences across breeds in adaptation to various environments and in performance on complex traits such as growth rate are very common in livestock. These differences have been attributed to various factors, including genetic variation, selection, and environmental influences. Gene expression regulation, serving as a critical intermediary mechanism that bridges genotypes and phenotypes, may play a pivotal role in driving these differences across breeds. Hence, we characterized the breed-shared and breed-specific pattern in genetic regulatory effects on gene expression via expression quantitative trait locus (eQTL) mapping in three pig breeds (Duroc, Landrace, and Yorkshire), aiming to gain a deeper understanding of the molecular basis underlying complex trait differences across breeds.

**Results:**

We observed breed differentiation at both the single-nucleotide polymorphism (SNP) and gene expression levels. By eQTL mapping, within each tissue, an average of 71.1% of the eGenes identified in each breed were breed-shared, while the remaining 28.9% were breed-specific. We found that some regulatory effects are relevant to either the difference in average gene expression or expression variance among populations. Breed-shared eGenes were more abundant, showed larger effect sizes and lower evolutionary conservation, and vice versa. Enrichment analysis showed that the genome-wide association studies (GWAS) loci were significantly enriched in the *cis*-eQTLs of eGenes for an average of 12 of 19 complex traits per breed. These loci exhibited higher enrichment for breed-specific eGenes than for breed-shared eGenes. Through colocalization analyses with GWAS loci, we observed 220 colocalization events (PP.H4 > 0.8) with breed-specific eGenes and 758 events with breed-shared eGenes.

**Conclusions:**

Our study reveals breed-shared and breed-specific effects and characteristics of genetic regulation on gene expression in three pig breeds. Both breed-shared and breed-specific eGenes contribute to the regulatory variation in complex traits, with breed-specific eGenes capturing additional regulatory signals not explained by breed-shared eGenes. Together, these findings demonstrate that both shared and breed-specific regulatory variation play important roles in shaping gene expression and suggest their potential contribution to complex traits.

**Supplementary Information:**

The online version contains supplementary material available at 10.1186/s40104-026-01374-2.

## Background

As the most common and important modern commercial pig breeds, Duroc, Landrace, and Yorkshire exhibit distinct differences in complex trait performance, including growth rate, meat quality, immune response, and reproductive capacity, reflecting their different breeding histories and selection goals. In the pig industry, the Duroc pig is one of the most widely used commercial boar lines and is well-known for its growth, feed conversion efficiency, carcass, and meat quality traits [1], which possess strong skeletal muscle growth potential and high lean meat yield [2]. In contrast, Landrace pigs are primarily employed as maternal lines in commercial breeding programs due to their superior reproductive performance, characterized by large litter size, strong maternal behavior, and high milk production [3]. Yorkshire pigs combine excellent growth performance, high lean meat yield, and strong adaptability, which are frequently crossed with Landrace pigs to produce F1 hybrid sows for enhanced reproductive and maternal traits [4, 5]. Intensive artificial selection has strongly targeted genes relating to production performance, reproduction, and carcass traits in these commercial breeds, thereby contributing to differences in complex traits across breeds [6]. A combination of genetic variation, selective breeding, and environmental factors influences this phenotypic diversity. Although genome-wide association studies have identified numerous loci associated with traits over the past decades, most significant GWAS loci are found in non-coding regions, and these genetic variants often fail to fully explain the observed phenotypic differences, suggesting that additional regulatory mechanisms may play a role in shaping traits [7].

Besides genetic variation, gene expression differences have also emerged as a key factor shaping complex traits. Gene regulation serves as a key intermediate layer that connects genotypes to phenotypes [8]. Past research showed that integrating genetic variation with gene expression patterns can help reveal the genetic basis of these molecular traits and their relationships with higher-order phenotypes [9]. Expression quantitative trait loci eQTLs are genomic variants that influence gene expression levels across individuals, and the genes they regulate are termed eGenes. Identifying eQTLs and eGenes enables functional interpretation of regulatory variants and provides a crucial link between genetic variation and phenotypic traits. With the establishment of large-scale transcriptomic resources such as cattleGTEx [10], pigGTEx [11], and FarmGTEx [9], integrative eQTL studies across multiple breeds in livestock have emerged, highlighting the importance of molecular traits, particularly gene expression regulation, in understanding the genetic basis of complex traits.

In livestock, most studies of molecular phenotypes have focused on a multi-breed design, aiming to identify genetic effects shared across breeds. However, different breeds can exhibit distinct patterns of complex traits, and approaches that primarily emphasize shared effects may overlook breed-specific regulatory mechanisms [12]. As a result, such studies may fail to capture the full spectrum of variants contributing to complex traits. Similarly, many human diseases differ among populations in prevalence, severity, or age of onset, and these differences can be partly explained by variation in gene expression regulation [13]. However, early studies were largely confined to populations of European ancestry, limiting the generalizability of their findings and potentially overlooking genetic effects specific to other populations [14]. More recent research incorporating diverse ancestral backgrounds has revealed that some regulatory effects are shared across ancestries, while others are ancestry-specific, demonstrating that ancestry-specific regulatory mechanisms can explain additional phenotypic variation beyond shared effects [15–17]. However, little is known about the shared and breed-specific regulatory effects across pig breeds.

To investigate breed-shared and breed-specific characteristics in gene expression regulation among pig breeds, we applied *cis*-eQTL mapping in Duroc, Landrace, and Yorkshire pigs. By integrating these results with genome-wide association studies, we further explored the contribution of breed-shared and breed-specific regulatory variation to phenotypic diversity. This work provides a preliminary exploration of breed-dependent genetic regulation and its contribution to complex traits.

## Methods

### Population and data

The population used in our study consisted of 300 pigs for 3 different breeds (100 Duroc, 100 Landrace, and 100 Yorkshire) generated from [18]. The genotypic data in VCF format and raw RNA-seq data in FASTQ format of this population were downloaded from the GigaScience GigaDB database (10.5524/102388). Animals from each breed were raised under standardized commercial farming conditions, with comparable housing and feeding, and were slaughtered at approximately six months of age. All individuals (*n* = 300) were genotyped using whole-genome sequence (WGS) with a depth of ~ 10 × . The downloaded genotypic data comprised 31,682,957 SNPs. We retained 15,495,927 biallelic variants with minor allele frequency (MAF) ≥ 5% and minor allele count (MAC) ≥ 6 in 18 autosomes after quality control across 300 pigs. To confirm the breed information of the 300 pigs, we used ADMIXTURE (v1.3.0) [19] to estimate individual ancestry proportions, following the breed predication analysis pipeline implemented in PigGTEx [11].

The raw RNA-seq data used in this study included three tissues, duodenum, muscle, and liver for each individual, resulting in a total of 900 RNA-seq samples. We used Trimmomatic (v0.39) [20] to trim adaptors and discard reads with poor quality, and then used STAR (v2.7.0) [21] to align clean reads to the Sscrofa11.1(v100) pig reference genome. Gene raw read counts were acquired with featureCounts (v1.5.2) [22], and normalized expression (transcripts per million, TPM) was derived from these counts. We retained 29,000 genes in 18 autosomes across 300 pigs.

### Differential gene expression analysis

We performed differential gene expression analysis between one breed and the other two breeds. We assessed expression differences across breeds within each tissue using the TPM data with limma (R package) [23]. We defined genes with |log_2_FC| > 1 and FDR < 0.05 as significantly differentially expressed genes.

### *Cis*-eQTL mapping

We performed *cis*-eQTL mapping in each tissue of each breed separately. Quality control of the genotype data in each breed was performed using the following criteria: MAF > 0.01, MAC > 6, and het < 0.99. After separate quality control, 6,521,645 SNPs common to all three breeds were retained for downstream analyses. For gene expression data, we first split the gene expression profiles (TPM data) by tissue and breed to obtain the TPM matrix for each tissue of each breed. Then we filtered the low-expressed genes in the TPM data for each tissue and breed, defining them as genes with a TPM ≤ 0.1 in more than 80% of the samples. After obtaining the filtered genes for each tissue of each breed, we normalized the gene expression across samples using the trimmed mean of M-value (TMM) method, implemented in edgeR [24], followed by inverse normal transformation of the TMM.

We performed *cis*-eQTL mapping using a linear mixed model implemented in OmiGA [25] to test associations between normalized gene expression levels and SNPs in the ± 1 Mb of the transcription start site (TSS) of target genes. To control for the effects of the remaining potential confounders, the principal components of the gene expression (TMM data) were used as covariates, using the function of OmiGA (–geno-pc-covar 0 –dprop-pc-covar 0.001). OmiGA would select the first *n* PCs as covariates when the increase in the proportion of variance explained by *n* + 1 PCs and *n* + 2 PCs was less than 0.1% of the first *n* PCs.

The linear mixed model is as follows:

$$\boldsymbol y={\boldsymbol X}_\alpha+{\boldsymbol s}_\alpha\beta_\alpha+{\boldsymbol g}_\alpha+\boldsymbol e,$$

where ***y*** is an *n* × 1 vector of normalized gene expression levels (TMM data), ***X*** is an *n* × *c* matrix of covariates, including a column of 1, with corresponding fixed effect *α*, ${\boldsymbol s}_{\boldsymbol\alpha}$ is an *n* × 1 vector of mean centered genotypes values at the variant being tested, coded as 0, 1, or 2 for the AA, Aa, and aa genotypes. ${\boldsymbol \beta}_\alpha$ is the variant’s genetic effect, ${\boldsymbol g}_\alpha$ is an *n* × 1 vector of total genetic effects with $\boldsymbol{g}_{\alpha}\sim \mathrm{N}(0,{\boldsymbol{G}}{\upsigma }_{\alpha}^{2})$ where the genomic relationship matrix (GRM) is defined as $\boldsymbol{G}=\frac{\boldsymbol{MM}^{\prime}}{2\sum_{i=1}^{m}{p}_{i}\left(1-{p}_{i}\right)}$ [26], of which ***M*** is a matrix of mean centered genotypes for genome-wide genetic variants and ${{p}}_{{i}}$ is the MAF of the *i*^th^ variant, ***e*** is *n* × 1 vector of residuals with $\boldsymbol{e}\sim \mathrm{N}(0, {\boldsymbol{I}}_{{n}}{\upsigma }_{\mathrm{e}}^{2})$ where ${\boldsymbol I}_n$ is an *n* × *n* identity matrix.

We considered SNPs with a nominal *P*-value below the variant-level threshold (obtained from permutations) as significant *cis*-eQTLs. We considered eGenes with a gene-level *P*-value corrected by the Benjamini–Hochberg method that was below the significance threshold (*q* value < 0.05).

### Definition and functional annotation of breed-shared and breed-specific eGenes

Breed-shared eGenes were defined as eGenes that exist in at least two breeds within the same tissue. Breed-specific eGenes were defined as eGenes that exist in only one breed within the same tissue. Breed-shared eGenes included two-breeds-shared eGenes (eGenes only shared in Duroc and Landrace, Duroc and Yorkshire, Landrace and Yorkshire) and three-breeds-shared eGenes (eGenes shared across Duroc, Landrace, and Yorkshire). To further resolve the potential biological functions of eGenes, we performed Gene Ontology (GO) and Kyoto Encyclopedia of Genes and Genomes (KEGG) pathway enrichment analysis on breed-shared eGenes and breed-specific eGenes using clusterProfiler (R package) [27].

### Sharing-pattern of effect size and direction

We applied the multivariate adaptive shrinkage (mashr**)** [28] to account for sparsity and correlations among effects of *cis*-eQTLs across breeds. We used the effect estimates and standard errors for *cis*-eQTLs of breed-shared eGenes across three breeds. We quantified effect sharing by both sign (same direction) and magnitude (similar size, within a factor of 2), and considered effects significant if the local false sign rate (LFSR) was < 0.05.

### Linkage disequilibrium score

We calculated linkage disequilibrium (LD) for breed-specific eGenes. For each breed, SNPs located within ± 1 Mb of the TSS of breed-specific eGenes were extracted, and LD scores were computed using GCTA [29] based on the squared correlation coefficient between pairs of SNPs.

### PhastCons score calculation of eGenes

PhastCons scores of 100 vertebrate species from UCSC (http://hgdownload.cse.ucsc.edu/goldenpath/hg38/phastCons100way/hg38.100way.phastCons/) were downloaded to use in our study. We first converted the Wiggle files of PhastCons scores to bed files using the BEDOPS tool (v2.4.40) [30], and then lifted over from human genome 38 (h38) to Sscrofa11.1 using UCSC’s LiftOver tool [31]. We used the mean PhastCons scores of sequences within a gene to represent its PhastCons score. Genes were considered only if at least 50% of their sequence length could be mapped in LiftOver.

### GWAS summary statistics

The GWAS summary statistics used in this study were generated from [32]. We used meta-GWAS summary statistics integrating multiple populations and breeds (mainly including Duroc, Landrace, and Yorkshire). Only GWAS summary statistics records containing at least one SNP with a *P*-value ≤ 5 × 10^−8^ were retained for downstream analyses. The traits utilized for enrichment and colocalization analysis include 19 traits, they were average daily gain (ADG, *n* = 36,943), backfat thickness (BFT, *n* = 58,725), body weight (BW, *n* = 42,256), days (DAYS, *n* = 49,595), gestation days (GD, *n* = 13,325), lean cuts percentage (LEANCUTP, *n* = 17,523), loin muscle area (LMA, *n* = 26,176), loin muscle depth (LMDEP, *n* = 34,439), number born alive (NBA, *n* = 13193), number born alive (day 21) (NBA_D21, *n* = 1,083), number born of healthy pigs (NBH, *n* = 17,746), number born of stillborn pigs (NBS, *n* = 11,521), total litter weight of piglets born alive (TLWT_BA, *n* = 19,701), total litter weight of piglets (day 21) (TLWT_D21, *n* = 9,064), total litter weight of piglets (weaning) (TLWT_Weaning, *n* = 11,166), total number of born (TNB, *n* = 22,217), teat number (TNUM, *n* = 38,158), uterine capacity (UC, *n* = 8,571), and weaning to estrus interval (WSI, *n* = 16,261).

### Enrichment analysis of eGenes and GWAS signals

We performed enrichment analysis between GWAS signals and the *cis*-eQTLs of eGenes. Significant GWAS SNPs with *P*-value ≤ 5 × 10^–8^ and the eGenes region defined as ± 1Mb of the TSS were used for the analysis. We defined significant GWAS SNPs as the observed set and randomly sampled an equal number of SNPS from the non-significant SNPs as a control set. This sampling procedure was repeated 1,000 times to generate a background distribution. The fold enrichment was then calculated as the proportion of significant GWAS SNPs located in eGene regions divided by the mean proportion of the proportion of control set falling within eGene regions.

### Colocalization of *cis*-eQTL and GWAS

We performed colocalization analysis between breed-shared, breed-specific eGenes and GWAS summary statistics using coloc (R package) [33]. Colocalization was performed for each eGene using SNPs within ± 1 Mb of its TSS. We considered colocalization to be significant when the posterior probability for a shared causal variant (PP.H4) exceeded 0.8.

## Results

### Population structure and gene expression patterns for three pig breeds

**Fig. 1:**
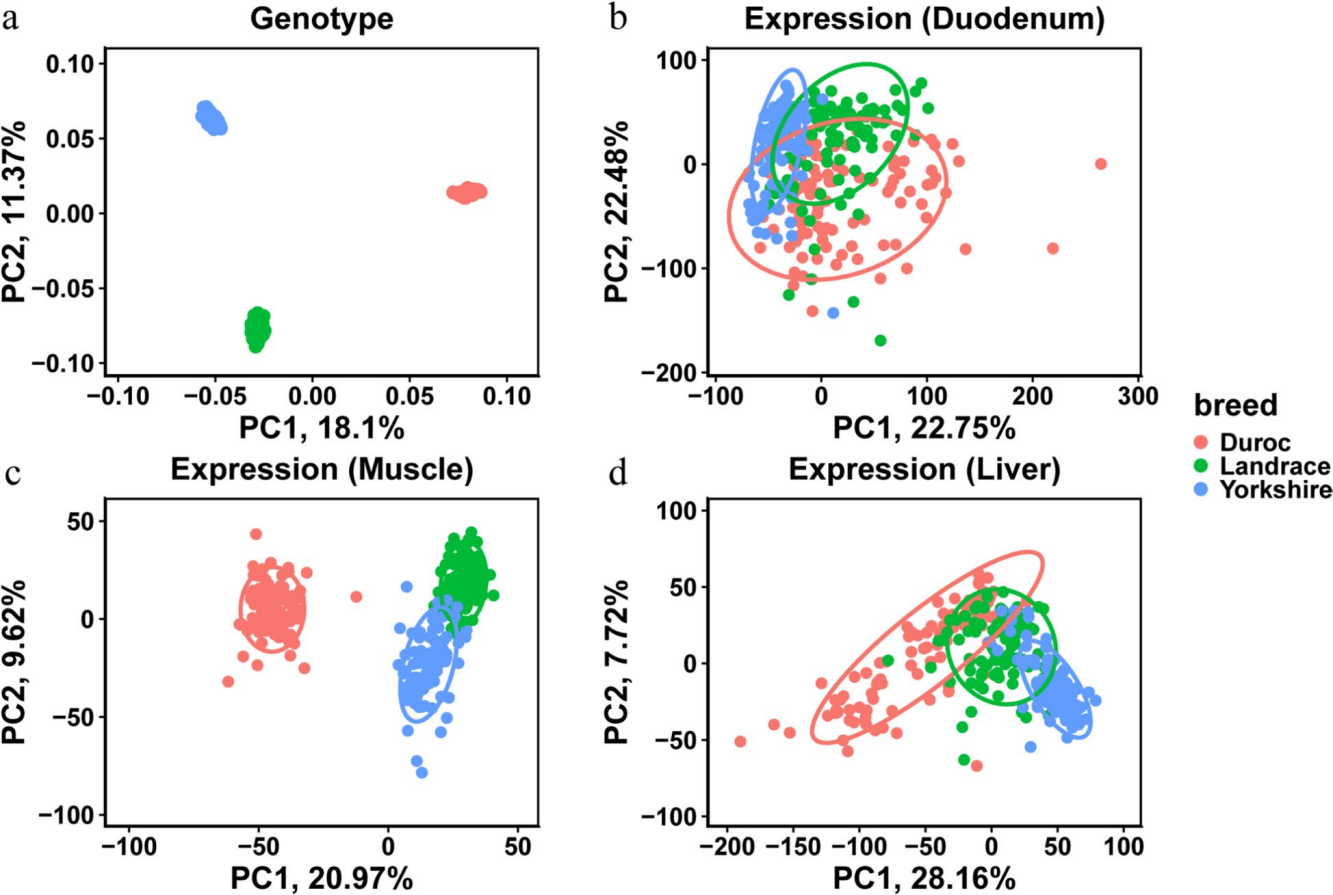
Clustering of genotype and gene expression data. **a** The PCA result of genotype data across three breeds. **b** The PCA result of 29,000 genes in the duodenum across three breeds. **c** The PCA result of 29,000 genes in muscle across three breeds. **d** The PCA result of 29,000 genes in the liver across three breeds

After quality control, we retained 6,521,645 common SNPs across three pig breeds (i.e., Duroc, Landrace, and Yorkshire). To explore the population structure of these pigs, we performed PCA using the genotypic data from all 300 individuals. We observed that these individuals clustered mainly according to breeds (Fig. 1a). The first and second genotype PCs (i.e., PC1 and PC2), explained 18.10% and 11.37% of the variance, respectively. To explore the patterns of gene expression among these three pig breeds, we calculated the Pearson’s correlation using gene expression levels from all 900 samples from three tissues. The heatmap of gene expression correlations between samples showed that samples from the same tissue exhibited higher correlation, especially within muscle and liver, indicating that different tissue types contribute more to gene expression variation than different breeds (Fig. S1). We further performed PCA in each of the three tissues based on the log-transformed TPM data from 29,000 genes. We observed that the samples tended to be clustered by different breeds, with Landrace and Yorkshire positioned closer to each other than either is to Duroc (Fig. 1b–d). Moreover, we identified the differentially expressed genes among the three breeds for each tissue. In the duodenum, muscle, and liver, we found that 867, 949, and 2,904 differentially expressed genes for Duroc, 351, 461, and 612 for Landrace, and 763, 282, and 612 for Yorkshire. Although breed differences at the genomic level and tissue differences at the transcriptomic level have been well established, our results indicate that breed-specific transcriptomic differences also exist within tissue.

### Breed-stratified *cis*-eQTL mapping and eGenes sharing patterns

**Fig. 2:**
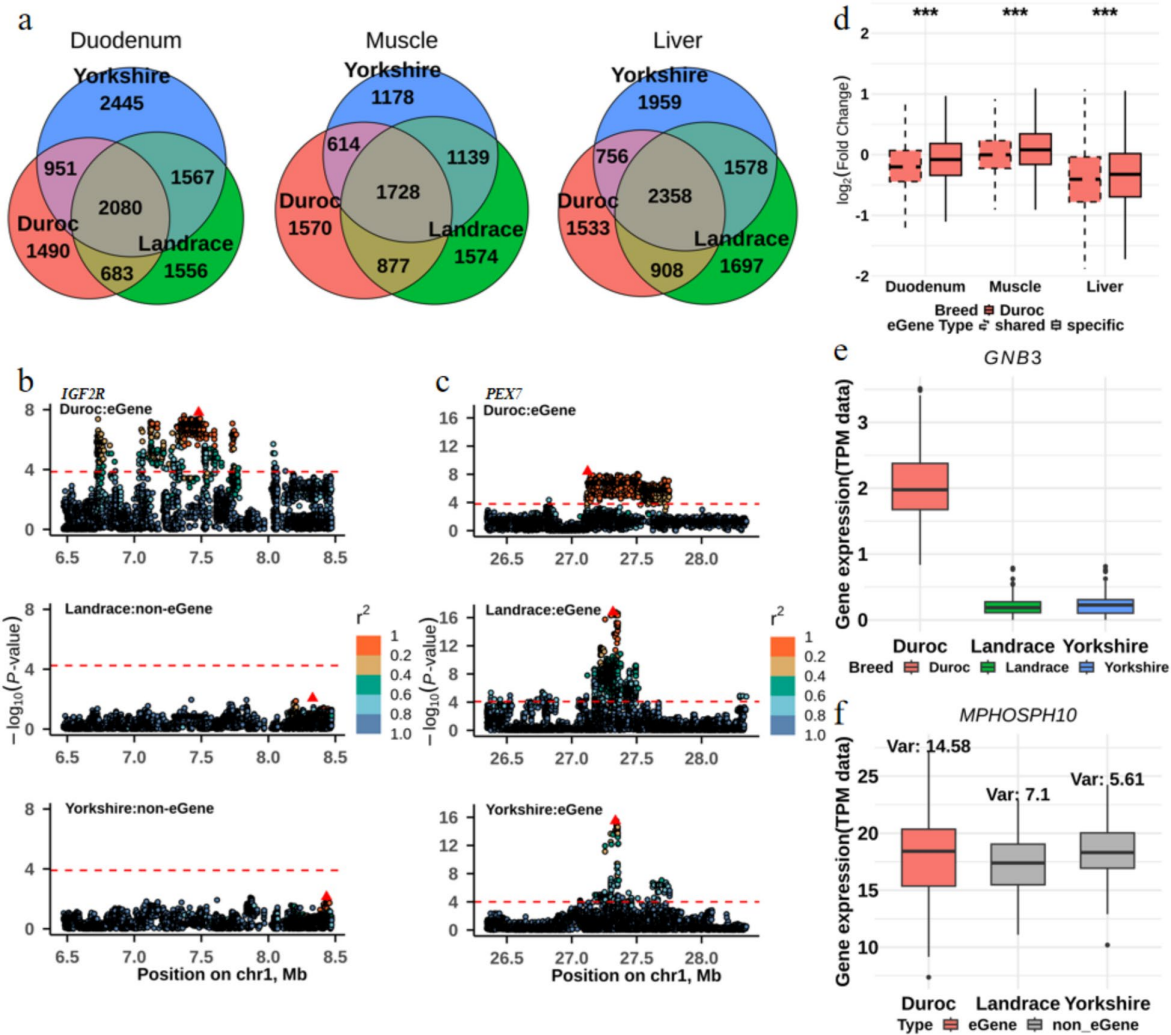
Breed-shared and breed-specific eGenes. **a** The number of eGenes in three tissues across three breeds. **b** Manhattan plot of a Duroc-specific eGenes *IGF2R* in muscle. **c** Manhattan plot of a breed-shared eGenes *PEX7* in muscle. **d** The fold change result of Duroc-shared eGenes and Duroc-specific eGenes (removed outliers, and significance test was performed using Mann–Whitney-Wilcoxon test, “ ∗ ” indicates *P* < 0.05, “ ∗ ∗ ” indicates *P* < 0.01, “ ∗ ∗ ∗ ” indicates *P* < 0.001). **e** The gene expression levels of *GNB3*, a differentially expressed gene and Duroc-specific eGene in Duroc muscle. **f** The gene expression levels of *MPHOSPH1*0 across three breeds (*MPHOSPH1*0 is a Duroc-specific eGene and a non-differentially expressed gene, and is not an eGene for Landrace and Yorkshire)

To investigate the contribution of genetic regulation to gene expression differences across pig breeds, we performed *cis*-eQTL mapping separately in the duodenum, muscle, and liver for each breed. We identified 5,204, 4,789, and 5,555 eGenes in the duodenum, muscle, and liver of Duroc, respectively; 5,886, 5,318, and 6,541 eGenes in Landrace; and 7,043, 4,659, and 6,651 eGenes in Yorkshire. We further classed them into breed-specific eGenes and breed-shared eGenes, of which breed-shared eGenes include eGenes shared in two or three breeds (Fig. 2a). Within each tissue, an average of 71.1% of the eGenes identified in each breed were breed-shared, while the remaining 28.9% were breed-specific. It indicated that the majority of *cis*-regulatory effects on gene expression are conserved across breeds, which is consistent with findings from the PigGTEx project [11] and some human studies [34–36]. Notably, Duroc shared fewer eGenes with the other two breeds in all tissues. Specifically, in the duodenum, 1,567 eGenes were shared between Landrace and Yorkshire, whereas Duroc shared only 683 eGenes with Landrace and 951 eGenes with Yorkshire. For instance, the Duroc-specific eGene *IGF2R* was involved in the mannose-6-phosphate (M6P) sorting pathway, which mediates the transport of phosphorylated lysosomal enzymes from the Golgi complex and the cell surface to lysosomes (Fig. 2b), and a breed-shared eGene *PEX7* was involved in Peroxisomal protein import and Ether lipid biosynthesis pathways (Fig. 2c). These results indicate that there are differences in regulatory effects for gene expression across three pig breeds.

### Characteristics of breed-shared and breed-specific eGenes

To investigate the characteristics of breed-shared and breed-specific eGenes, we compared the fold change values from differential expression analysis between breed-shared and breed-specific eGenes. We found that Duroc-specific eGenes exhibit significantly higher expression specificity in the Duroc population than the other two breeds (Fig. 2d). For example, the *GNB3* gene is a Duroc-specific eGene that exhibits a high average expression level in Duroc (Fig. 2e). We also observed some breed-specific eGenes like *MPHOSPH10* that show large expression variation (Fig. 2f). Similar patterns were observed in muscle for the Landrace population, muscle and liver for the Yorkshire population (Fig. S2). These results indicate that breed-specific regulatory effects influence not only differences in average gene expression but also variability in expression among populations.

To investigate the potential biological functions of these breed-shared and breed-specific eGenes, we performed GO and KEGG pathway enrichment analyses. We observed that the breed-shared eGenes in muscle were significantly enriched in carboxylic acid metabolic process (fold enrichment = 1.64, FDR < 0.05), and other biological processes (Additional file 1: Table S1 and S2). The Duroc-specific eGenes in muscle were enriched in biological processes such as regulation of mitotic cell cycle (fold enrichment = 2.67, FDR < 0.05) and negative regulation of gene expression (fold enrichment = 1.98, FDR < 0.05; Additional file 1: Table S3 and S4).

**Fig. 3:**
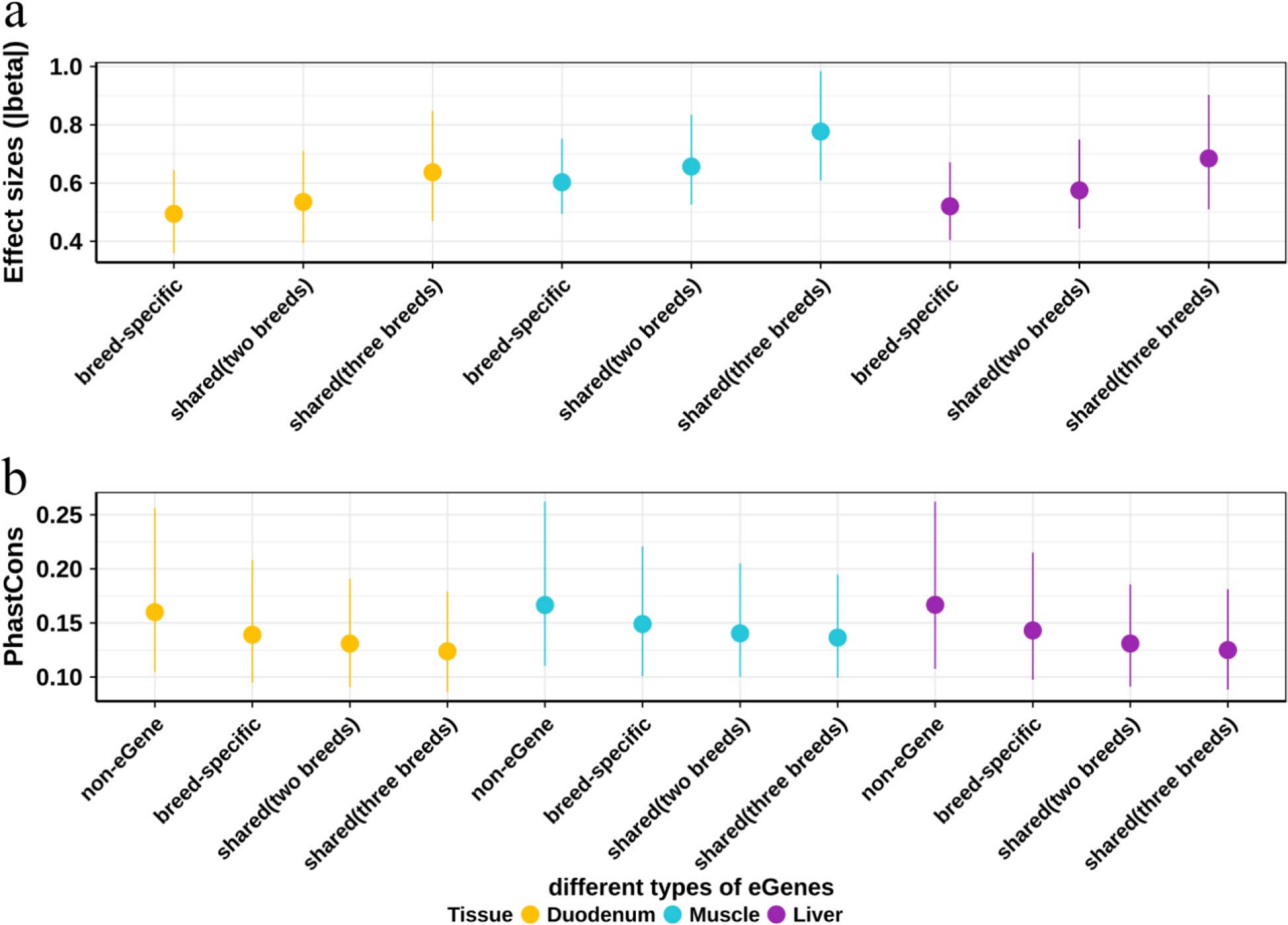
Effect sizes and PhastCons scores of different types of eGenes. **a** Effect sizes of lead *cis*-eQTLs for eGenes. Breed-specific eGenes included Duroc-, Landrace-, and Yorkshire-specific eGenes. Shared (two breeds) eGenes were those shared between Duroc and Landrace, Duroc and Yorkshire, and Landrace and Yorkshire, excluding those shared among all three breeds. Shared (three breeds) eGenes were those shared across Duroc, Landrace, and Yorkshire. **b** PhastCons score of eGenes. Non-eGenes were genes that were not identified as eGenes in any of the three breeds. Other types of eGenes were the same in Fig. 3a

To further investigate the genetic regulatory effects and evolutionary conservation of breed-shared and breed-specific eGenes, we examined the effect sizes (beta) of their lead *cis*-eQTLs and their PhastCons score. We found that the lead *cis*-eQTLs exhibit higher regulatory effect sizes for breed-shared eGenes than breed-specific eGenes (Fig. 3a). For instance, in muscle, the median effect sizes were 0.60 for breed-specific eGenes, 0.66 for eGenes shared in two breeds, and 0.78 for eGenes shared in three breeds. From the PhastCons score, an evolutionary conservation across 100 vertebrates, we observed that eGenes shared across three breeds present the lowest cross-species conservation than those eGenes only detected in one breed or not detected in any breeds (Fig. 3b). For instance, in liver, the median effect sizes were 0.17 for non-eGenes, 0.14 for breed-specific eGenes, 0.13 for eGenes shared in two breeds, and 0.12 for eGenes shared in three breeds. These findings suggest that breed-shared eGenes have stronger regulatory effect sizes and lower evolutionary conservation, whereas breed-specific eGenes have smaller effect sizes and higher evolutionary conservation. Similarity patterns have also been reported in human studies [37, 38].

To further quantify the extent of regulatory effect sharing across breeds, we assessed pairwise sharing of eQTL effects underlying breed-shared eGenes. Under both stringent sign and magnitude sharing criteria (within a twofold difference), the estimated effect sharing proportions ranged from 0.19 to 0.27 across breeds in all three tissues, indicating equivalent effect across breeds (Fig. S3). Notably, sharing between Landrace and Yorkshire was consistently higher than that involving Duroc, broadly concordant with their closer genetic relationship observed in the PCA analysis. We also compared the sharing proportion based on direction concordance along, and direction with a broader magnitude threshold (within a fourfold difference). We observed that a large proportion of effects underlying breed-shared eGenes exhibited consistent directions across breeds, ranging from 0.86 to 0.89. When the broader magnitude threshold was applied, the sharing proportion increased from 0.19–0.27 to 0.79–0.84**,** indicating that many effects share the same direction but differ in effect size across breeds. These results suggest that the regulatory effects of breed-shared eGenes often have concordant directions, while the magnitude of these effects can vary across breeds.

To assess differences in genetic background associated with breed-specific regulatory signals, we compared local LD structure and allele frequency patterns of lead SNPs of breed-specific eGenes across three breeds. The distributions of LD scores differed across breeds, with Duroc showing a higher median LD score and a broader high-LD tail, and Landrace tended to exhibit lower median LD scores (Fig. S4). These results indicate breed differences in local LD patterns. In addition, the lead SNPs of breed-specific eGenes showed clear differences in MAF across breeds. For each breed-specific eGene, the lead SNPs consistently had the highest median MAF in the breed in which the eGene was detected, while the other two breeds exhibited similar and generally lower median MAF values (Fig. S5). These results suggest that breed-specific regulatory variations were associated with differences in local LD structure and MAF across breeds.

### Contribution of breed-stratified *cis*-eQTLs for complex traits of pigs

**Fig. 4:**
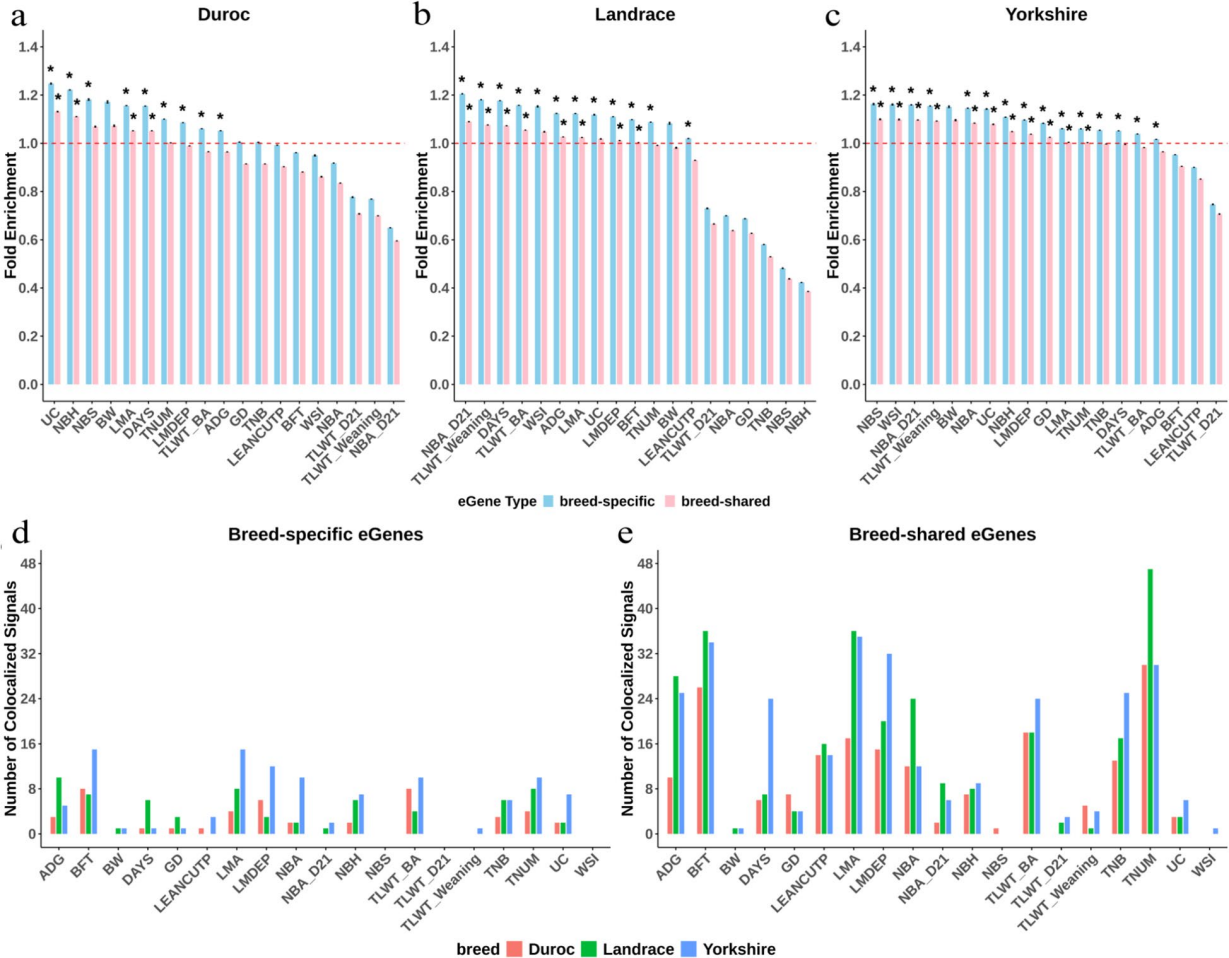
The result of GWAS signal enrichment and colocalization analyses. **a** The fold enrichment result of Duroc-shared and Duroc-specific eGenes. **b** The fold enrichment result of Landrace-shared and Landrace-specific eGenes. **c** The fold enrichment result of Yorkshire-shared and Yorkshire-specific eGenes (“ ∗ ” indicates *P* < 0.05). **d** The significant colocalization result of breed-specific eGenes. **e** The significant colocalization result of breed-shared eGenes

To evaluate the contribution of breed-shared and breed-specific eGenes to the regulation of pig complex traits, we performed the enrichment analysis using GWAS summary statistics for significant associations from 19 complex traits. We observed that GWAS loci were significantly enriched in the *cis*-eQTLs of eGenes on average across 12 of 19 complex traits per breed in Duroc, Landrace, and Yorkshire. Notably, these GWAS loci exhibited higher enrichment in breed-specific eGenes than breed-shared eGenes (Fig. 4a–c). This result suggests that, similar to the *cis*-eQTLs of breed-shared eGenes, these *cis*-eQTLs of breed-specific eGenes play a crucial role in regulating the complex traits of pigs.

To explore whether causal SNPs are shared between breed-shared and breed-specific eGenes and GWAS signals for pig complex traits, we performed colocalization analysis to identify shared regulatory effects between gene expression and complex traits. Totally, we observed 758 and 220 colocalization events (PP.H4 > 0.8) with breed-shared and breed-specific eGenes, respectively. The colocalization events from these *cis*-eQTLs of breed-shared eGenes are substantially higher than those of *cis*-eQTLs of breed-specific eGenes (Fig. 4d and e). The more observations of colocalization events on breed-shared eGenes than breed-specific is consistent with the observations on the number of eGenes.

**Fig. 5:**
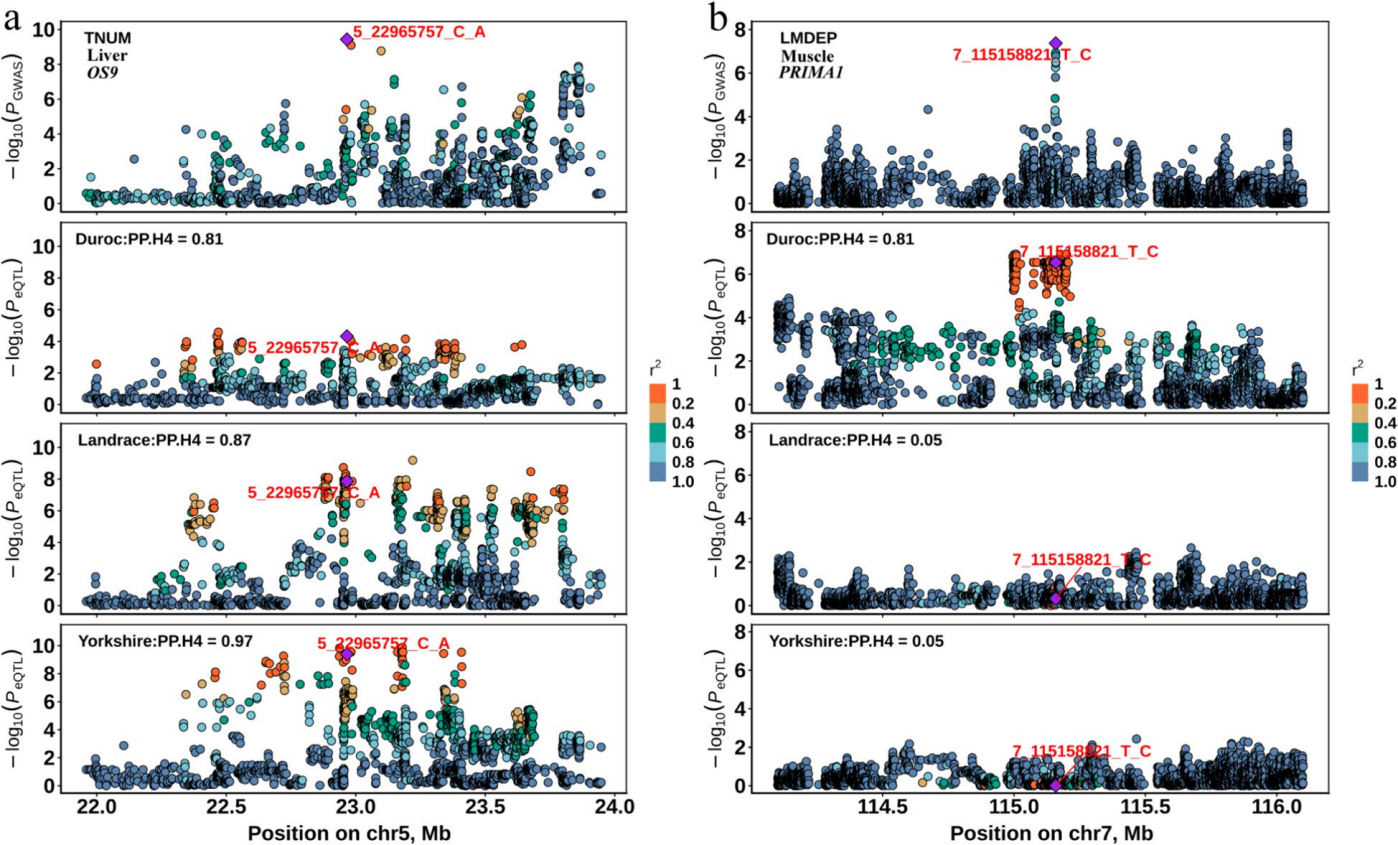
The colocalization examples of breed-shared eGenes and breed-specific eGenes. **a** A breed-shared eGene *OS9* on chr5 was significantly colocalized with the TNUM trait across Duroc, Landrace, and Yorkshire. **b** A breed-specific eGenes *PRIMA1* on chr7 was significantly colocalized with the LMDEP trait in Duroc, and was not significantly colocalized with Landrace and Yorkshire

For example, there is a GWAS signal in chromosome 5 of the TNUM trait colocalized with the *cis*-eQTLs of the *OS9* gene in liver across three pig breeds (Fig. 5a), of which the *OS9* is a breed-shared eGene in liver across three breeds. *OS9* encodes a lectin component of the mammalian HRD1 ubiquitin ligase complex, which is essential for multiple physiological processes, including metabolic regulation, maintenance of intestinal homeostasis, immune cell function, prohormone maturation, and β-cell identity [39]. In humans, *OS9* has been reported as a stable housekeeping gene in breast tissue and has also been implicated in breast cancer biology [40]. While our findings suggest a potential link between *OS9* regulation in the liver and TNUM variation across three pig breeds, the precise molecular mechanisms underlying this association remain to be elucidated.

The GWAS signal of the LMDEP trait colocalized with the *cis*-eQTLs of the *PRIMA1* gene in muscle, of which the *PRIMA1* gene is a Duroc-specific eGene in muscle (Fig. 5b). The function of the *PRIMA1* gene is to organize acetylcholinesterase (AChE) into tetramers and to anchor AChE at neural cell membranes. A previous study demonstrated that *PRiMA*-linked G4 acetylcholinesterase (AChE) is localized at neuromuscular junctions (NMJs) and that its expression in motor neurons contributes to this synaptic localization [41], suggesting a critical role in regulating muscle contraction. *PRIMA1* expression is also influenced by neuronal differentiation and maturation [42], which may contribute to variation in its expression across breeds. Our colocalization analysis suggests that breed-specific regulation of *PRIMA1* may contribute to variation in this muscle-related trait. These findings highlight that, through complementarity, the *cis*-eQTLs of breed-specific eGenes could explain additional GWAS signals for complex traits.

## Discussion

Understanding the genetic regulation of gene expression across different breeds is crucial for interpreting the molecular basis of phenotypic diversity and complex traits. In this study, we systematically identified breed-shared and breed-specific eGenes across three pig breeds and assessed their potential contributions to complex traits through enrichment and colocalization analyses, highlighting that both shared and breed-specific regulation variation jointly contribute to gene expression and complex traits.

Breed-shared and breed-specific eGenes exhibited distinct regulatory features. Breed-shared eGenes accounted for the majority of *cis*-regulatory effects, and their regulatory directions were highly consistent, indicating that most gene regulation was conserved across breeds. Shared eGenes also exhibited larger effect sizes but lower evolutionary conservation, suggesting that they may regulate general cellular and metabolic processes that can tolerate regulatory variability without detrimental consequences [37]. Breed-specific eGenes capture regulatory variation unique to each breed, characterized by smaller effect sizes and higher conservation than breed-shared eGenes. Domestication and long-term artificial selection have led to genetic divergence among the three breeds [43–45], including differences in LD and allele frequencies. The breed-specific regulatory variation we observed inherently reflects these breed-specific genomic features, including differences in LD and MAF. Our breed-stratified analyses therefore capture regulatory variation within each breed’s native genetic context.

Our study demonstrated that the majority of eGenes were shared across breeds, which were consistent with observations in other species [11, 37, 46]. We analyzed 100 individuals per breed with a balanced design, and the smaller effect sizes of breed-specific eQTLs likely limited the detection given our sample size. Genotype PCA revealed the significant genetic differences among breeds. Although the overall separation among breeds for breed-specific differences was detectable at the transcriptomic level within tissue, this separation was more subtle than at the genomic level. At the transcriptome level, breed separation is not very obvious. Together with the broadly conserved *cis*-regulatory effects, this may explain the high proportion of shared eGenes (71.1%) across breeds. In addition, we applied a stringent false discovery rate threshold (FDR < 0.05) and consistent criteria across breeds to ensure that the identified eGenes represent statistically robust regulatory signals. Consistent with previous studies, shared eGenes are generally defined based on overlap across populations.

Some eGenes were differentially expressed, while a subset of the remaining eGenes had higher expression variance in the specific breed, indicating that genetic variants may influence expression variability rather than mean expression. This concept aligns with variance QTLs, which capture regulatory effects on phenotypic variability and uncover additional regulatory signals, particularly gene–environment interactions [47–49]. Applying similar approaches in pigs may uncover breed-specific regulation not detected by standard eQTL mapping.

Both breed-shared and breed-specific eGenes colocalized with complex traits (Fig. 4d and e), indicating that both shared and breed-specific regulatory effects on gene expression contribute to complex traits. Similar findings in human studies, where ancestry-specific eQTLs provide additional explanatory power for complex traits [16]. Consistently, our findings further highlight the contribution of breed-specific regulatory variation to phenotypic variation in pigs. Duroc pigs exhibited marked genetic and transcriptional divergence from Landrace and Yorkshire, with numerous Duroc-specific eGenes in each tissue. In muscle, Duroc-specific eGenes were enriched in biological processes related to cell differentiation and proliferation. Importantly, these eGenes were also associated with growth traits (e.g., DAYS, LMDEP, and LMA), as evidenced by significant enrichment in GWAS loci and colocalization signals with growth traits. These results suggest that Duroc-specific regulatory variation in gene expression may influence muscle growth by modulating cellular differentiation and proliferative processes. Previous studies have reported that Duroc pigs possess a stronger myogenic potential, characterized by higher cellular activity and regenerative capacity in muscle development, and ligand–receptor interaction analyses of muscle stem cells revealed that Duroc myogenic lineage cells receive more proliferative signals [50]. Together, these findings suggest that Duroc-specific regulatory effects on genes involved in muscle cell differentiation and proliferation may underlie breed-dependent differences in growth rate and muscle traits. Nevertheless, while colocalization analyses provide evidence for shared genetic signals between gene expression and complex traits, they do not by themselves establish causality. Future studies should integrate genetic variation, GWAS results, context-specific multi-omics (e.g., ATAC-seq, ChIP-seq, Hi-C, single-cell RNA-seq), and functional validations to gain a deeper understanding of the genetic architecture underlying breed-shared and breed-specific regulation.

The original study of the dataset performed a joint-breed expression GWAS and did not specifically address breed-stratified regulatory variation [18]. Such studies may mask breed-specific regulatory variation. Through our breed-stratified analysis, we identified 3,791, 3,781, and 4,469 breed-specific eGenes in the duodenum, muscle, and liver that were not detectable in the joint-breed analysis. Approximately 93.5% of the expressed genes identified in the original study were also detected in our analysis, highlighting overall concordance while revealing additional breed-specific regulatory variation. This study enabled us to investigate both breed-shared and breed-specific aspects of gene regulation. It is well established that the power of eQTL mapping is correlated to the sample size [7]. In this study, 100 individuals per breed may limited statistical power and reduce the ability to detect regulatory effects with small effect sizes, and thus lead to an underestimation of the full extent of regulatory variation. Future efforts incorporating larger sample sizes, additional tissues, and a broader range of pig breeds, together with models that account for multi-breed interaction terms, will provide a more appropriate framework for assessing regulatory variation across breeds. These approaches will facilitate a more comprehensive assessment of shared and breed-specific regulatory architecture and improve our understanding of the genetic basis of phenotypic divergence.

## Conclusions

Our study reveals both breed-shared and breed-specific patterns of genetic regulation on gene expression across pig breeds. Consistent with previous findings, gene regulation was broadly conserved. Breed-shared eGenes were abundant and tended to have larger effect sizes and lower evolutionary conservation, and breed-specific eGenes showed smaller effect sizes and higher conservation. Both types of eGenes contribute to complex trait variation, with breed-specific eGenes capturing additional regulatory effects. These results highlight a layered regulatory architecture that should be taken into account in multi-breed studies of gene regulation and in the genetic dissection of complex traits, enhancing our understanding of breed-stratified regulatory architecture and its contribution to complex traits.

## Supplementary Information

Additional file 1: Table S1. The GO result of breed-shared eGenes. Table S2. The KEGG result of breed-shared eGenes. Table S3. The GO result of breed-specific eGenes. Table S4. The KEGG result of breed-specific eGenes.

Additional file 2: Fig. S1. The Person’s correlation of gene expression levels. Fig. S2. The fold change result of Landrace and Yorkshire eGenes. Fig. S3. Pairwise sharing of cis-eQTL effects for breed-shared eGenes across three pig breeds. Fig. S4. LD score distribution of the lead SNPs of breed-specific eGenes. Fig. S5. Comparison of MAF of lead SNPs for breed-specific eGenes across breeds.

## Data Availability

The genotype data and RNA-Seq data are obtained from http://dx.doi.org/10.5524/102388 and the SRA Accession: PRJEB58031.

## Abbreviations

AchE: Acetylcholinesterase
ADG: Average daily gain
BFT: Backfat thickness
BW: Body weight
eQTL: Expression quantitative trait locus
GWAS: Genome-wide association studies
GRM: Genomic relationship matrix
GO: Gene Ontology
GD: Gestation length
Het: Heterozygosity rate
KEGG: Kyoto Encyclopedia of Genes and Genomes
LD: Linkage disequilibrium
LEANCUTP: Lean cuts percentage
LFSR: Local false sign rate
LMA: Loin muscle area
LMDEP: Loin muscle depth
Mashr: Multivariate adaptive shrinkage
MAF: Minor allele frequency
MAC: Minor allele count
M6P: Mannose-6-phosphate
NMJs: Neuromuscular junctions
NBA: Number born alive
NBA_D21: Number born alive (d 21)
NBH: Number born of healthy pigs
NBS: Number born of stillborn pigs
PCA: Principal component analysis
SNP: Single-nucleotide polymorphism
TPM: Transcript per million
TMM: Trimmed mean of M-value
TSS: Transcription start site
TLWT_BA: Total litter weight of piglets born alive
TLWT_D21: Total litter weight of piglets (d 21)
TLWT_Weaning: Total litter weight of piglets (weaning)
TNB: Total number of born
TNUM: Teat number
UC: Uterine capacity
WGS: Whole genome sequencing
WSI: Weaning to estrus interval
