# Supplementary material for: Characterizing breed-shared and breed-specific genetic regulatory effects of gene expression across three pig breeds
Source: J Anim Sci Biotechnol. 2026 Apr 9;17:62. doi: 10.1186/s40104-026-01374-2 (PMC13063976; doi:10.1186/s40104-026-01374-2)
Supplement: Supplementary file 2 — Additional file 2: Fig. S1. The Person’s correlation of gene expression levels. Fig. S2. The fold change result of Landrace and Yorkshire eGenes. Fig. S3. Pairwise sharing of cis-eQTL effects for breed-shared eGenes across three pig breeds. Fig. S4. LD score distribution of the lead SNPs of breed-specific eGenes. Fig. S5. Comparison of MAF of lead SNPs for breed-specific eGenes across breeds. [file 40104_2026_1374_MOESM2_ESM.docx]

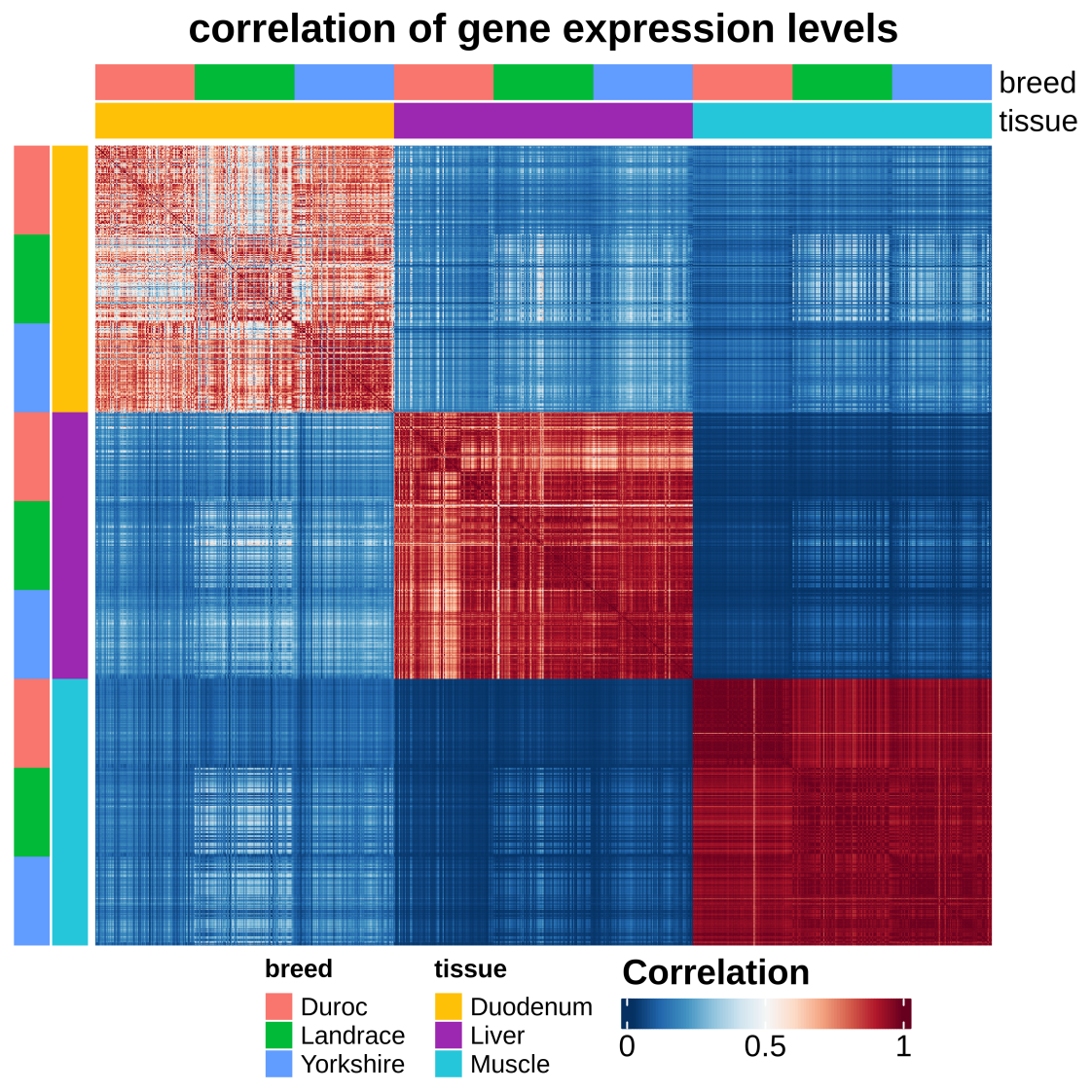


**Fig. S1.** The Person’s correlation of gene expression levels.


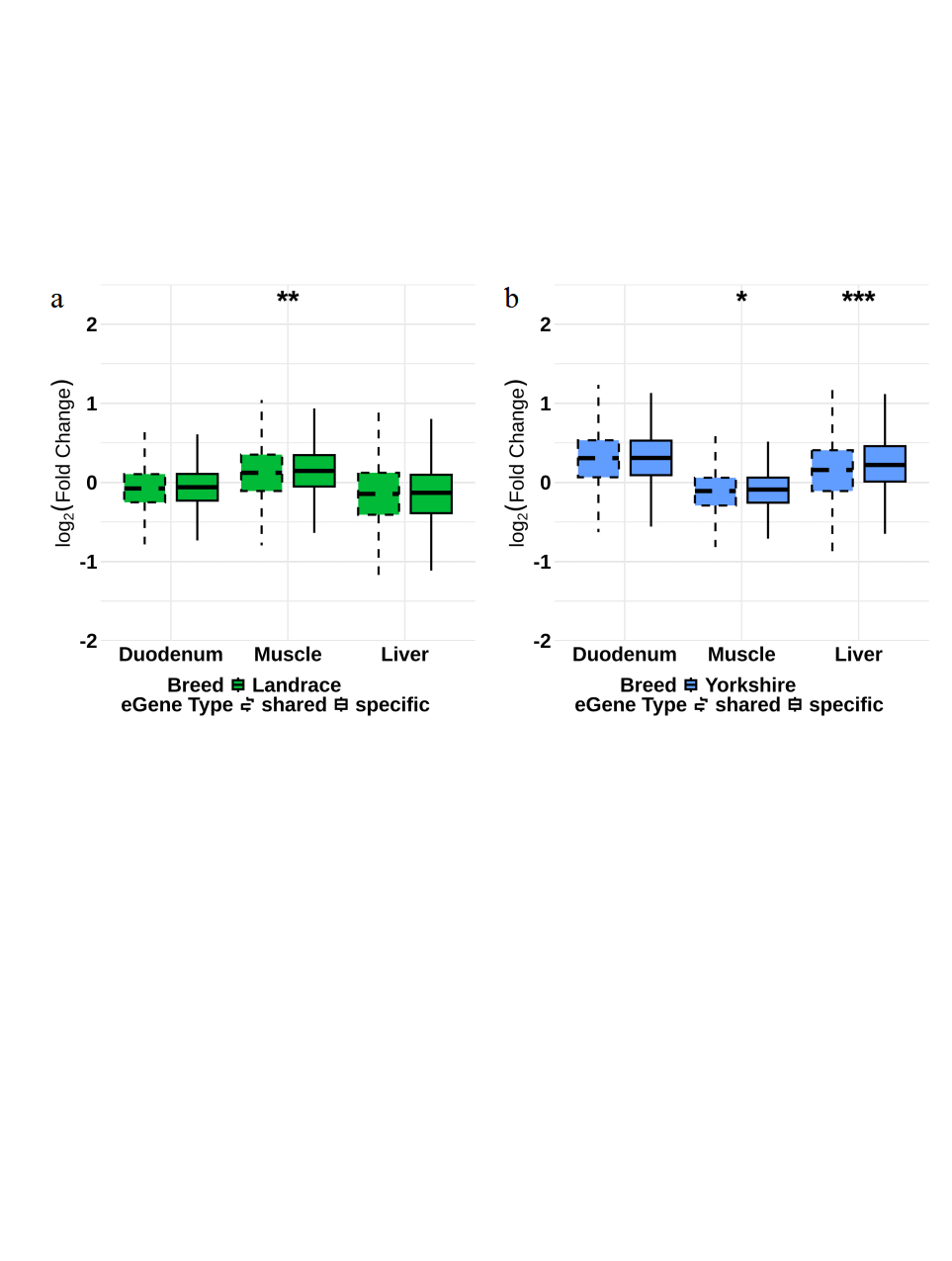


**Fig. S2.** The fold change result of Landrace and Yorkshire eGenes. a. The fold change result of Landrace-shared eGenes and Landrace-specific eGenes. b. The fold change result of Yorkshire-shared eGenes and Yorkshire-specific eGenes. (removed outliers, significance test was performed using Mann-Whitney-Wilcoxon test, “∗” indicates *P*< 0.05; “∗∗” indicates *P*< 0.01; and “∗∗∗” indicates *P*< 0.001)

##
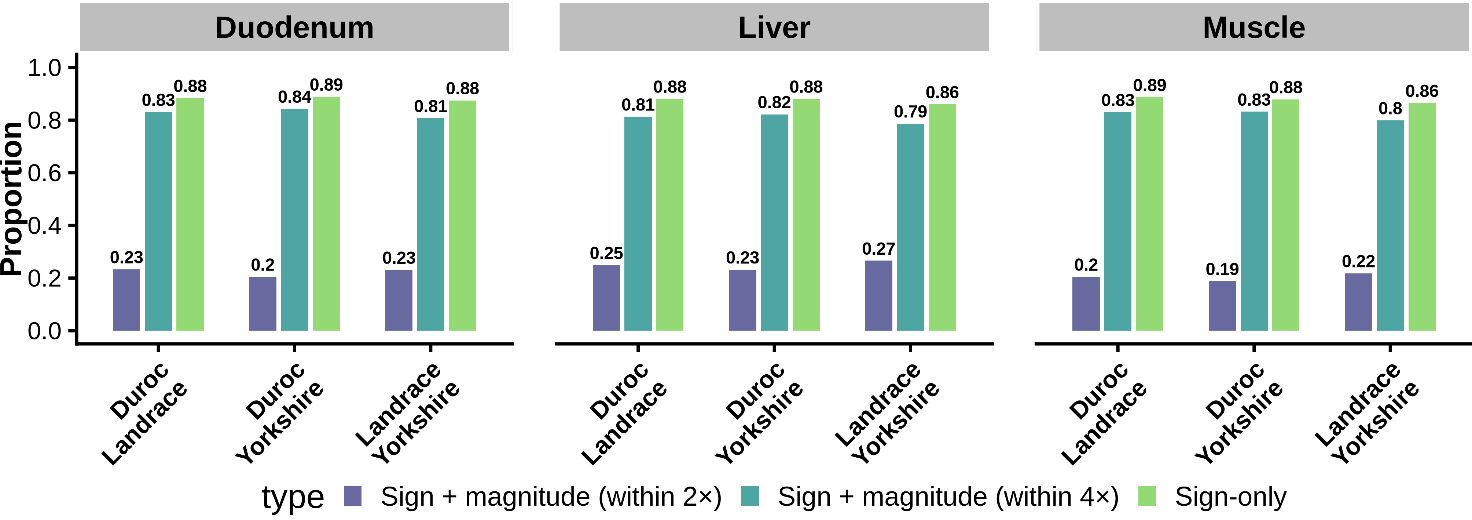


**Fig. S3.** Pairwise sharing of *cis*-eQTL effects for breed-shared eGenes across three pig breeds. Sharing proportions were estimated using mashr. “Sign + magnitude (within 2×)” refers to effects with the same direction and effect sizes within a twofold difference. “Sign + magnitude (within 4×)” refers to effects with the same direction and effect sizes within a fourfold difference. “Sign-only” refers to the direction concordance alone.


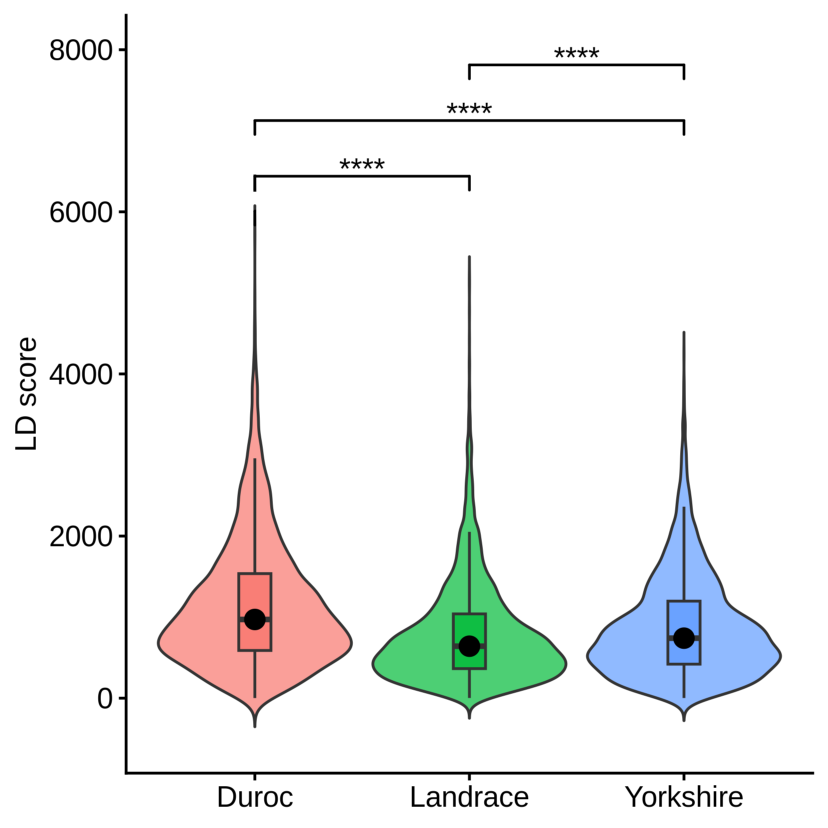


**Fig. S4.** LD score distribution of the lead SNPs of breed-specific eGenes. Violin plots show the distribution of LD scores calculated within the *cis*-regulatory regions of breed-specific eGenes for each pig breed. Statistical significance was assessed using the Wilcoxon test: “∗∗∗∗” indicates *P*< 0.0001.


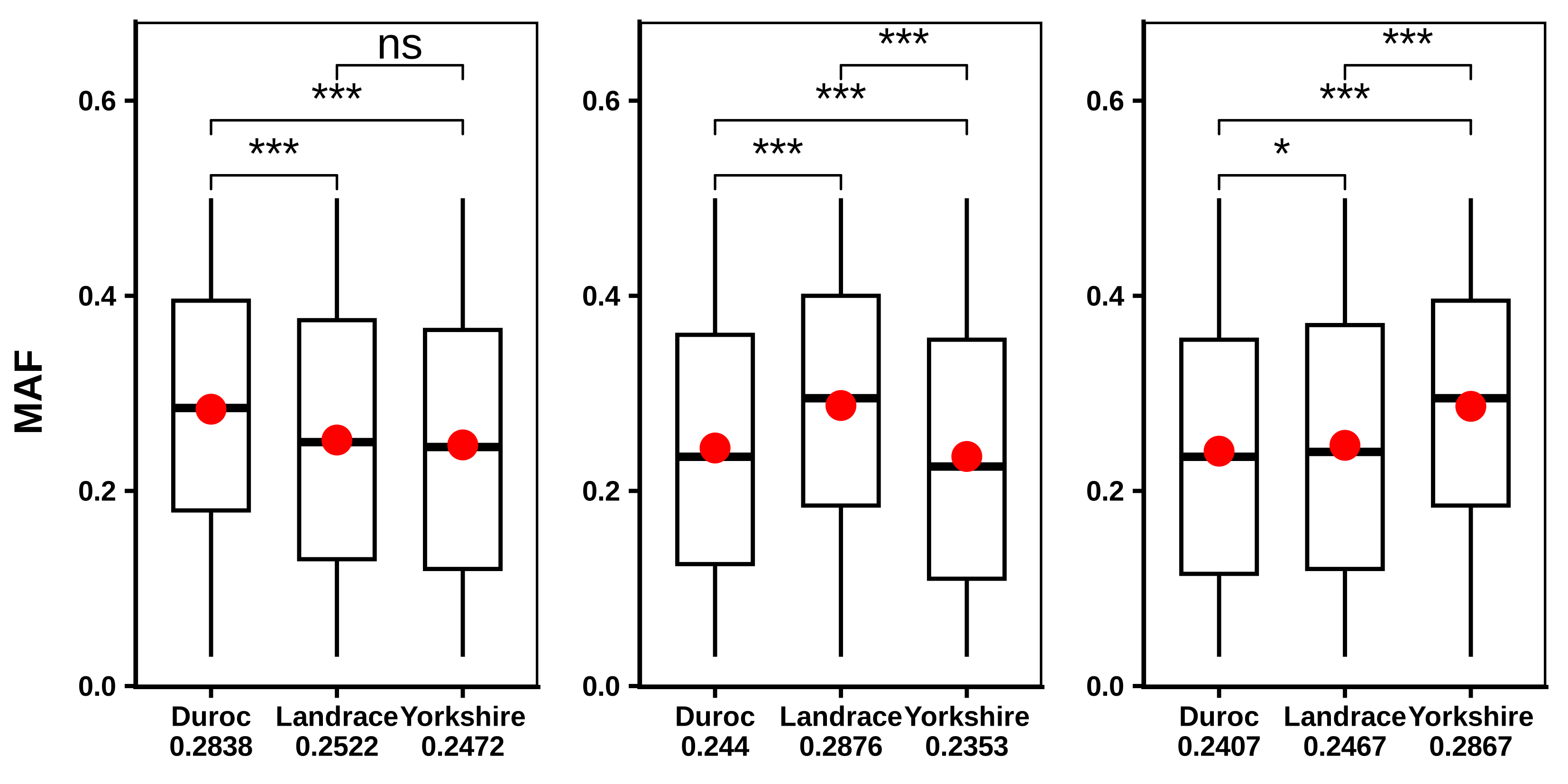


**Fig. S5.** Comparison of MAF of lead SNPs for breed-specific eGenes across breeds. MAF distributions of lead SNPs for breed-specific eGenes. From left to right, panels correspond to Duroc-specific, Landrace-specific, and Yorkshire-specific eGenes. Red points indicate mean MAF values, which are also labeled below the x-axis. Statistical significance was assessed using the Wilcoxon test: “ns” indicates not significant; “∗” indicates *P*< 0.05; “∗∗” indicates *P*< 0.01; and “∗∗∗” indicates *P*< 0.001.
